# Conservative management of a perianal rhabdomyosarcoma in a 2-year old child by Papillon’s technique

**DOI:** 10.1186/s13014-015-0413-9

**Published:** 2015-05-01

**Authors:** Charlotte Demoor-Goldschmidt, Sophie Dumoucel, Christine Haie-Meder, Nadège Corradini, Marc-André Mahé, Stéphane Supiot

**Affiliations:** Department of Radiotherapy, Institut de Cancérologie de l’Ouest- René Gauducheau, Bd J Monod, 44800 Nantes, St-Herblain France; Faculty of Medicine, University of Nantes, Nantes, France; Department of Pediatric Oncology, Institut Gustave Roussy, 39, rue Camille Desmoulins, 94805 Villejuif, France; Department of Brachytherapy, Institut Gustave Roussy, 39, rue Camille Desmoulins, 94805 Villejuif, France; Department of Pediatric Oncology, Centre Hospitalier Universitaire, Place Ricordeau, Nantes, France

**Keywords:** Rhabdomyosarcoma, Brachytherapy, External beam radiation therapy, Anus, Sphincter preservation

## Abstract

Rhabdomyosarcoma (RMS) is the most common sarcoma in paediatric patients. A perianal site is unusual and is associated with a low cure rate. The few cases of reported perianal RMS have been associated with sequelae. Here, we report the case of a 29-month-old male child who received sequential treatment by surgery, chemotherapy and radiotherapy inspired by Papillon’s irradiation of adult anal/low-rectum cancers (including external beam radiotherapy in the gynecological exam position followed by brachytherapy) and who remains in complete remission 49 months post treatment with no sphincter or other anorectal disorders.

Rhabdomyosarcoma (RMS) is the most common sarcoma in pediatric patients. A perianal site is unusual and is associated with high risk and a low cure rate [1]. The treatment of choice for RMS combines intensive chemotherapy, high-dose radiotherapy and complete surgical excision, but there is no established treatment strategy for RMS of the perineum or anus, in particularly in the Intergroup Rhabdomyosarcoma Staging (IRS) reports, as these locations are rare. The few cases of perianal RMS that have been reported have been associated with frequent sphincter disorders or anal ulcerations [2]. Wide first-line curative surgery is possible but causes loss of sphincter function. Conservative complete or partial surgery combined with adjuvant chemotherapy and/or radiotherapy may be considered in Stage 1 disease, care being taken to avoid dermatitis that might cause problems on subsequent radiotherapy [2]. This option helps spare sphincter function without jeopardizing oncological outcome.

However, a cogent multimodal therapeutic approach as used for anal or lower rectal carcinomas can secure sphincter preservation. In other situations (prostate, bladder or utero-vaginal), conservative RMS treatment has been shown to be possible by combining brachytherapy with surgery [3,4]. Here, we report the case of a 29-month-old male child who received sequential treatment by surgery, chemotherapy and a radiotherapy technique inspired by Papillon’s irradiation of adult anal and low-rectum cancers [5] (including external beam radiotherapy (EBRT) in the gynecological exam position followed by brachytherapy) and who remains in complete remission 49 months post treatment with no sphincter or other anorectal disorders.

## Case report

A 29-month-old male with no predisposing personal or family history was referred to the Surgery Department after his mother noticed a pararectal enlargement on bathing her child. MRI revealed a well-delineated heterogeneous left perianal mass (18×32×32 mm) with probable invasion of the external sphincter (no visible fatty interface), which was removed by finger-assisted enucleation. Pathology identified an alveolar RMS with PAX3–FKHR fusion transcript. No residual mass was visible on MRI. Left perianal hypermetabolism was observed on ^18^FDG PET-CT with no evidence of disease extension. A selective dissection of ilioinguinal lymph nodes visible on the MRI was performed and the pathologic analysis revealed no metastasis. According to the IRS grouping system, this tumor was classified as group III because of the incomplete initial surgery, therefore necessitating chemotherapy and adjuvant irradiation. Secondary surgery was avoided because it may have impaired the anal sphincter function. The patient received 9 cycles of chemotherapy (ifosfamide, vincristin, actinomycin over 2 days every 3 weeks during 7 months) without experiencing any severe adverse events.

The objective of the irradiation treatment was to deliver the equivalent of 50 Gy to the tumor bed which was defined as the Gross Tumor Volume (GTV) on the initial MRI. Two margins of 1 cm was applied to obtain respectively the Clinical Target Volume (CTV) and the Planning Target Volume (PTV) with retrieving 0.4 cm from the skin (posterior limit). The treatment position was thought to minimize irradiation to the femoral heads. Therefore, we decided to combine EBRT and brachytherapy similarly to the radiotherapy regimen pioneered by Papillon [6]. EBRT (28.8 Gy in 16 Gy fractions) was delivered during the 5th cycle (without actinomycin). Brachytherapy started 3 weeks and 5 days after the last session of EBRT and a few days before the 7th cycle (also without actinomycin).

**Figure 1:**
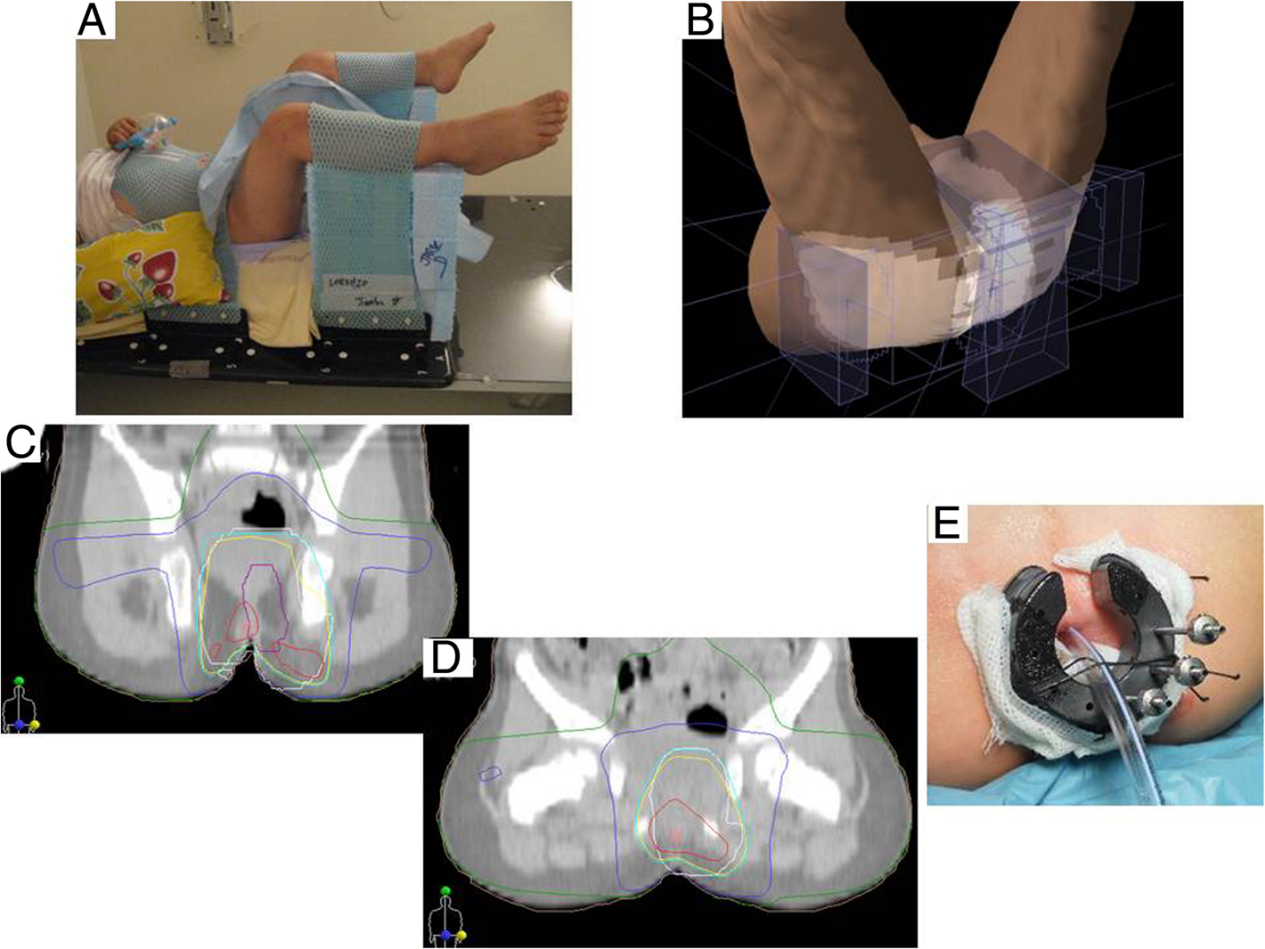
Radiation therapy. **(A)** patient positioning for EBRT using custom molded thermal plastic masks; **(B)** ballistics of the 4 treatment beams: 2 lateral beams and 2 direct perineal beams; **(C and D)** dosimetric illustration of the EBRT treatment (green 4 Gy, blue 10 Gy, cyan 20.58 Gy (95%), yellow 21 Gy, red 22.05 (105%); **C**: coronal incidence through the femoral heads; **D**: coronal incidence through the anal spincter **(E)** low dose rate brachytherapy (I192) with 3 needles implanted in initial tumor bed.

For EBRT, the child was placed in the gynecological exam position [Figure 1A] and immobilized using custom molded thermal plastic masks without any general anesthesia since the child was very compliant. EBRT was delivered with 6 MV photon beams: two direct radiation fields (an oblique anterior perineal-sacral field and a more posterior field at a 65° angle) and two lateral fields [Figure 1B]. This configuration reduced the radiation dose to healthy tissue and thus damage to the anus and femoral heads. We explored alternative techniques such as Intensity Modulated Radiation Therapy, but this option was not chosen to avoid low dose distribution to surrounding perineal tissues. EBRT was well tolerated without skin or intestinal toxicity.

One month after, low dose rate Iridium-192 interstitial brachytherapy (specific activity 5.17 μGy/h/m/cm,) was delivered. Needles were manually loaded with iridium wires of 35 mm length under general anesthesia. The technique delivered 21.2 Gy dose in a 5.5 cm^3^ volume according to the Paris system rules corresponding to the initial volume of the tumor visible on the MRI [Figure 1C]. The child was hospitalized during 4 days and did not require anesthesia during the brachytherapy application. Symptomatic treatment was delivered to manage the discomfort of the child and no adverse effect occurred during the brachytherapy application.

The maximum dose (EBRT + brachytherapy) to the left and right testicles was 8 Gy (7 + 1) and 1.5 Gy (1 + 0.5), respectively. Mean dose to the bladder was 29 Gy (28 + 1) and to femoral heads was only 12 Gy.

Overall treatment tolerance, whether gastrointestinal, cutaneous or mucosal, was satisfactory in the short and medium term. Toilet training was not delayed. Height-weight growth was normal. The child is still in complete remission 49 months after treatment, and presents no sphincter disorder or major local sequelae except limited telangiectasia in the brachytherapy boost region [Figure 1D]. Long-term follow-up will be required to monitor remission and detect any late complications.

## Discussion

According to the Intergroup RMS Study Group (IRSG) review of 71 children with perineal or anal RMS from 1972 through to 1997, the prognosis is poor, with a 5-year failure-free survival rate of only 45% and overall survival (OS) rate of 49% [4]. No details of the management of the radiation therapy are available. The lack of guidelines is a real difficulty to treat this rare disease. Median patient age was 6 years, 36% of patients were misdiagnosed at the time of surgical management, 45% had an initial biopsy diagnosis, and 64% had advanced stage disease at initial presentation. Delayed RMS diagnosis jeopardizes the chances of a cure [7,8]. A tumor size less than 5 cm was associated with a better 5-year OS rate (74% vs 37%).

The challenge for this child was to cure him while sparing his sphincter. A combination of radiotherapy techniques including EBRT and brachytherapy according to the sphincter preservation protocol pioneered by Jean Papillon at the Centre Léon Bérard (Lyon, France) for carcinomas of the lower rectum and anal canal [5,9,10] was easily delivered because of his excellent cooperation. Papillon treated carcinomas of the lower rectum and anal canal, with sphincter preservation, by conventional EBRT (30 Gy by a fixed direct perineal Cobalt-60 beam to the anus and the pelvic lymphatic drainage areas and 18 Gy by a posterior arc beam with a patient in the gynecological position). With this method, EBRT was followed after 2 months rest by Iridium-192 brachytherapy that delivered 20 Gy to a limited volume at a dose-rate of about 10 Gy/day by implanting in the anal sphincter 5 to 7 needles through a crescent-shaped template sutured to the perineal skin. His multimodal treatment was one of the first strategies to suggest that sphincter preservation in low gastrointestinal tract tumors was achievable with good clinical outcomes.

## Conclusion

Perianal RMS are high-risk tumors. Multimodal treatment should be initiated without delay. If possible, aggressive and mutilating first-line surgery should be avoided as positive surgical margins are common and postoperative sequelae can hamper subsequent treatment. Multimodal treatment may preserve sphincter function and achieve remission without major complications, and should not be withheld because of young age. If parents cooperate and all steps are clearly explained to the child, a patient positioning best suited to EBRT dosimetry can be used and low-dose rate brachytherapy (nowadays replaced by pulsed dose-rate brachytherapy) can be delivered over the course of a few days.

## Consent

Written informed consent was obtained from the parents of the patient for publication of this Case report and any accompanying images. A copy of the written consent is available for review by the Editor-in-Chief of this journal.
